# Reducing *Campylobacter jejuni* colonization in broiler chickens by in-feed supplementation with hyperimmune egg yolk antibodies

**DOI:** 10.1038/s41598-019-45380-z

**Published:** 2019-06-20

**Authors:** Jasmien Vandeputte, An Martel, Stefano Canessa, Nathalie Van Rysselberghe, Lieven De Zutter, Marc Heyndrickx, Freddy Haesebrouck, Frank Pasmans, An Garmyn

**Affiliations:** 1 Department of Pathology, Bacteriology and Avian Diseases, Faculty of Veterinary Medicine, Ghent University, Salisburylaan 133, B9820 Merelbeke, Belgium; 2 Department of Veterinary Public Health and Food Safety, Faculty of Veterinary Medicine, Ghent University, Salisburylaan 133, B9820 Merelbeke, Belgium; 3 Flanders Research Institute for Agriculture, Fisheries and Food (ILVO), Technology & Food Sciences Unit, Brusselsesteenweg 370, B9090 Melle, Belgium

**Keywords:** Public health, Zoology, Vaccines, Bacterial infection

## Abstract

*Campylobacter* infections sourced mainly to poultry products, are the most important bacterial foodborne zoonoses worldwide. No effective measures to control these infections in broiler production exist to date. Here, we used passive immunization with hyperimmune egg yolks to confer broad protection of broilers against *Campylobacter* infection. Two novel vaccines, a bacterin of thirteen *Campylobacter jejuni* (*C*. *jejuni*) and *C*. *coli* strains and a subunit vaccine of six immunodominant *Campylobacter* antigens, were used for the immunization of layers, resulting in high and prolonged levels of specific immunoglobulin Y (IgY) in the hens’ yolks. In the first *in vivo* trial, yolks (sham, bacterin or subunit vaccine derived) were administered prophylactically in the broiler feed. Both the bacterin- and subunit vaccine-induced IgY significantly reduced the number of *Campylobacter-*colonized broilers. In the second *in vivo* trial, the yolks were administered therapeutically during three days before euthanasia. The bacterin IgY resulted in a significant decrease in *C*. *jejuni* counts per infected bird. The hyperimmune yolks showed strong reactivity to a broad representation of *C*. *jejuni* and *C*. *coli* clonal complexes. These results indicate that passive immunization with hyperimmune yolks, especially bacterin derived, offers possibilities to control *Campylobacter* colonization in poultry.

## Introduction

Campylobacteriosis is one of the most important foodborne bacterial diseases worldwide and has been the most commonly reported zoonosis in the EU since 2005^1^. Clinical symptoms such as fever and diarrhoea are usually self-limiting, although in rare cases complications can occur, leading to reactive arthritis^2^, Guillain-Barré syndrome (GBS)^3^ and inflammatory bowel disease (IBD)^4^. The disease is mainly caused by *Campylobacter jejuni* (*C*. *jejuni*) and *Campylobacter coli* (*C*. *coli*)^1^ and contaminated chicken meat is considered a major source of infection^5^. Worldwide, over 50% of poultry meat is contaminated with *Campylobacter*^6^. However, no effective measures to limit *Campylobacter* infections in primary broiler chicken production exist to date^7^. Once a chicken is infected, the pathogen rapidly spreads infecting almost 100% of the flock within a week^8^.

Interestingly, chickens are only colonized from the age of two to three weeks onwards^9,10^, which is presumably due to the protection by maternal IgY antibodies (MAB)^11–13^. These antibodies are transferred from the serum of the mother to the egg yolk, protecting the chicks during the first weeks when their immune system is not yet fully developed^13^. From two weeks onward, the blood concentration of MAB against *Campylobacter* drops significantly, which coincides with an increased colonization susceptibility of the chickens. As a measure, pure MAB or egg yolks of immunized chickens containing pathogen specific MAB can be added to the feed of the chicks to prolong this effect^13,14^. Previously, Hermans *et al*.^15^ immunized laying hens with a whole-cell lysate of *C*. *jejuni* or its hydrophobic protein fraction, and successfully used their eggs to protect young chickens against *Campylobacter* infection. As such, passive immunization of broiler chickens using egg yolk IgY offers possibilities to control *C*. *jejuni* colonization in broiler flocks.

The vaccines tested by Hermans *et al*.^15^ were based on one single *C*. *jejuni* strain, which is not representative for the field situation with many genetically different strains^16^. A bacterin containing heterogeneous *Campylobacter* strains might offer a much broader target reactivity. Also, Hermans *et al*.^15^ identified several immunodominant *C*. *jejuni* antigens. A subunit vaccine containing a mix of broadly conserved, immunodominant proteins could lead to a well-defined and standardized vaccine.

We developed two vaccines to immunize laying hens against *C*. *jejuni* and *C*. *coli* to obtain IgY-rich eggs that confer broad protection of chickens against *C*. *jejuni* and *C*. *coli* infection: a bacterin consisting of genetically heterogeneous *Campylobacter* strains relevant to the field situation and a subunit vaccine containing multiple recombinant immunodominant antigens of *C*. *jejuni* strain KC40^15^. Egg yolks of hens immunized with these vaccines were used for passive oral immunization of broiler chickens to investigate their prophylactic and therapeutic efficacy against experimental *Campylobacter* infection in broiler chickens. Finally, the reactivity of these egg yolks to a variety of *C*. *jejuni* and *C*. *coli* strains, belonging to different clonal complexes was tested as a proxy for the breadth of protection.

## Results

### Immunodominant antigens are highly prevalent and highly conserved in *C*. *jejuni*

A PCR analysis, amplifying AtpA, Ef-Tu, GroEL, Tig, CheV and LivJ encoding gene fragments, resulted in positive PCR products in every *C*. *jejuni* strain screened. Sequence analysis of the PCR products and translation of the nucleotide sequences into protein sequences showed conservation levels of 97–100% for both gene and protein sequences (sequence data published elsewhere^17^). Screening the *C*. *coli* strains, positive PCR products were only obtained for LivJ, CheV and Ef-Tu with conservation levels of 80%, 96% and 99%, respectively, for both gene and protein sequences (sequence data published elsewhere^17^).

### Preparation of recombinant *C*. *jejuni* antigens

**Figure 1:**
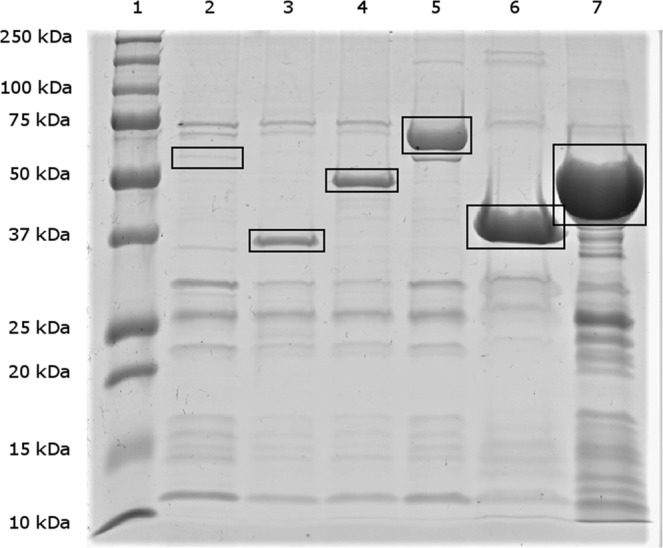
SDS-PAGE analysis visualized by Brilliant Blue G-Colloidal coloring of recombinant *C*. *jejuni* proteins. Column 1: protein marker with size labelling in kilodalton (kDa) at the left, 2: AtpA (54.8 kDa), 3: CheV (35.8 kDa), 4: EfTu (43.6 kDa), 5: GroEL (58.0 kDa), 6: LivJ (40.1 kDa), 7: Tig (51.0 kDa).

Gene copies of *C*. *jejuni* KC40 AtpA, Ef-Tu, GroEL, Tig, CheV and LivJ were cloned successfully in an entry vector and the pDEST™17 destination vector and expressed in BL21-AI One Shot® *E*. *coli* transformants. Results of the SDS-PAGE analysis of recombinant *C*. *jejuni* antigens are shown in Fig. 1. All proteins were detected at their corresponding length.

### Immunization of layers with the bacterin and subunit vaccine dramatically induces *Campylobacter*-specific egg yolk IgY titers

The bacterin- and subunit vaccine-induced *Campylobacter-*specific IgY titers in the egg yolks, determined by ELISA, are given in Tables 1 and 2. These yolk titers were maintained for at least two years after final immunization.

**Table 1:** Bacterin- and subunit vaccine-induced egg yolk IgY titers against the bacterin and individual *Campylobacter* strains used in this study, as determined by ELISA.

Strain	Antibody titers induced by	
	Bacterin	Subunit
Bacterin	1:65,536	1:16,384
KC40^b^	1:65,536	1:16,384
10kf-1.16^b^	1:65,536	1:512
7P6.12^b^	1:65,536	1:16,384
10C-6.1^b^	1:65,536	1:16,384
10kf-4.12^b^	1:65,536	1:65,536
10VTDD-8^b^	1:65,536	1:16,384
T124^b^	1:65,536	1:16,384
T84^b^	1:65,536	1:65,536
T70^b^	1:65,536	1:65,536
2012/3291^b^	1:65,536	1:2,048
5970^b^	1:65,536	1:16,384
2013/2711^b^	1:65,536	1:16,384
2012/3250^b^	1:65,536	1:32,768
5CT13	1:65,536	1:4,096
3CT13	1:32,768	<1:32
1CT117	1:32,768	1:32
1CT51	1:32,768	<1:32

^b^strains incorporated in the bacterin.

**Table 2:** Bacterin- and subunit vaccine-induced egg yolk IgY titers against the subunit vaccine and its individual antigen compounds, as determined by ELISA.

Antigen	Antibody titers induced by	
	Bacterin	Subunit
Subunit	1:256	1:65,536
AtpA	1:512	1:32,768
CheV	<1:32	1:32,768
EfTu	1:512	1:32,768
GroEL	<1:32	1:32,768
LivJ	1:128	1:32,768
Tig	1:128	1:32,768

The bacterin-induced IgY titers against the bacterin and the different *Campylobacter* bacterin strains were all 1:65,536. Also against the *Campylobacter* strains belonging to different clonal complexes (CC) than the bacterin *Campylobacter* strains, IgY titers were remarkably high (1:32,768 to 1:65,536). The subunit vaccine-induced IgY titer against the bacterin was 1:16,384. The subunit vaccine-induced IgY titers against the different *Campylobacter* bacterin strains varied from 1:65,536 (10kf-4.12, T84, T70) to 1:512 (10kf-1.16). For the *Campylobacter* strains belonging to different CC’s than the bacterin *Campylobacter* strains, a strong reaction was observed for one strain only (1:4,096; 5CT13). For the subunit vaccine-induced IgY antibodies, a titer of 1:65,536 was obtained against the subunit vaccine and titers of 1:32,768 against each recombinant antigen, separately. The bacterin-induced IgY antibodies showed a much lower reaction, with titers varying from 1:512 (AtpA, EfTu) to non-detectable (<1:32; CheV, GroEL) against the separate recombinant antigens and a titer of 1:256 against the subunit vaccine.

### Prophylactic passive immunization of broilers with bacterin and subunit vaccine derived hyperimmune egg yolk significantly reduces the number of *C*. *jejuni* colonized birds

In the first *in vivo* trial, the prophylactic effect of hyperimmune egg yolks from immunized laying hens administered to the feed of broiler chickens was investigated. *C*. *jejuni* counts per gram (g) cecal content after euthanasia of the chickens are summarized in Table 3. Posterior distributions of the estimated probabilities of *C*. *jejuni* colonization and mean colonization load are added as Supplemental Fig. S1. Both the number of *Campylobacter*-positive birds and the mean *C*. *jejuni* titers of these positive birds should be considered when interpreting the data about the global *Campylobacter* populations.

**Table 3:** Number of positive birds and mean cecal *C*. *jejuni* counts of colonized broilers receiving standard feed supplemented with 5% (wt/wt) egg yolk from either bacterin-immunized, subunit vaccine-immunized or sham-immunized (control) layers, from day 1 until day 16 (the day of euthanasia).

	Number of positive birds			Mean *C*. *jejuni* counts of positive birds		
	Bacterin	Subunit	Control	(log_10_(cfu/g cecal content)) (*Standard deviation*)		
				Bacterin	Subunit	Control
**All birds**						
Group 1	2/9	4/9	5/9	4.48 (*0*.*25*)	3.64 (*2*.*18*)	3.81 (*1*.*53*)
Group 2	2/9	4/9	8/9	3.50 (*0*.*71*)	5.71 (*1*.*83*)	4.31 (*1*.*74*)
Group 3	0/9	4/9	8/9	- (*−*)	5.08 (*1*.*88*)	4.59 (*1*.*80*)
Treatment	**4/27** ^**a**^	**12/27** ^**a**^	**21/27** ^**b**^	**3**.**99**^**a**^ (***0***.***71***)	**4**.**81**^**a**^ (***2***.***00***)	**4**.**30**^**a**^ (***1***.***66***)
**Seeders**						
Group 1	0/3	3/3	3/3	- (*−*)	3.85 (*2*.*62*)	4.39 (*1*.*60*)
Group 2	1/3	2/3	3/3	4.00 (*−*)	6.01 (*2*.*39*)	5.76 (*2*.*09*)
Group 3	0/3	1/3	3/3	- (*−*)	5.04 (*−*)	4.81 (*2*.*31*)
Treatment	**1/9** ^**a**^	**6/9** ^**ab**^	**9/9** ^**b**^	**4**.**00**^**a**^ (*−*)	**4**.**77**^**a**^ (***2***.***24***)	**4**.**99**^**a**^ (***1***.***85***)
**Sentinels**						
Group 1	2/6	1/6	2/6	4.48 (*0*.*25*)	3.00 (*−*)	2.94 (*1*.*33*)
Group 2	1/6	2/6	5/6	3.00 (*−*)	5.41 (*2*,*00*)	3.45 (*0*.*81*)
Group 3	0/6	3/6	5/6	- (*−*)	5.10 (*2*.*30*)	4.46 (*1*.*72*)
Treatment	**3/18** ^**a**^	**6/18** ^**a**^	**12/18** ^**b**^	**3**.**98**^**a**^ (***0***.***87***)	**4**.**85**^**a**^ (***1***.***94***)	**3**.**79**^**a**^ (***1***.***38***)

At 11 days of age, 3 seeder birds per group were inoculated with approximately 10^5^ cfu *C*. *jejuni* KC40. A random effect was included in the statistical model at the subgroup (pen) level to account for clustering.

The total number of *C*. *jejuni* colonized broilers in the groups receiving hyperimmune egg yolk from bacterin-immunized layers (4/27) and subunit vaccine-immunized layers (12/27) was significantly lower than the number of *C*. *jejuni* colonized birds in the control subgroups (21/27; resp. *p* = 0.0030 and *p* = 0.041), or a reduction from 78% to resp. 15% and 44% infected birds. This also applies for the seeder birds separately (resp. 1/9, 6/9 and 9/9; *p* = 0.00056 and *p* = 0.025). For the sentinels, hyperimmune egg yolk from bacterin-immunized layers but not from subunit vaccine–immunized layers significantly reduced the number of *C*. *jejuni* colonized broilers compared to the control broilers (resp. 3/18, 6/18, 12/18; *p* = 0.022 and *p* = 0.088). The treatments did not significantly differ from each other. No significant differences were observed for the *C*. *jejuni* counts in birds positive for colonization.

### Therapeutic passive immunization of broilers with bacterin derived hyperimmune egg yolk significantly reduces cecal *C*. *jejuni* titers

In the second *in vivo* trial, the therapeutic potential of hyperimmune egg yolk from immunized laying hens administered to the feed of broiler chickens to reduce cecal *C*. *jejuni* colonization was assessed. *C*. *jejuni* counts per g cecal content after euthanasia of the chickens are summarized in Table 4. Posterior distributions of the estimated probabilities of *C*. *jejuni* colonization and mean colonization load are added as Supplemental Fig. S2.

**Table 4:** Number of positive birds and mean cecal *C*. *jejuni* counts of colonized broilers receiving standard feed supplemented with 5% (wt/wt) egg yolk from either bacterin-immunized, subunit vaccine-immunized or sham-immunized (control) layers, from day 19 until day 21 (the day of euthanasia).

	Number of positive birds			Mean *C*. *jejuni* counts of positive birds		
				(log_10_(cfu/g cecal content)) (*Standard deviation*)		
	Bacterin	Subunit	Control	Bacterin	Subunit	Control
**Group 1**	7/9	9/9	7/9	3.12 (*1*.*31*)	5.56 (*1*.*28*)	5.64 (*1*.*80*)
**Group 2**	8/9	9/9	7/9	4.08 (*1*.*59*)	5.19 (*1*.*84*)	4.96 (*2*.*09*)
**Group 3**	7/9	8/9	6/9	4.74 (*1*.*03*)	5.55 (*1*.*34*)	5.10 (*2*.*11*)
**Treatment**	**22/27** ^**a**^	**26/27** ^**a**^	**20/27** ^**a**^	**4**.**00**^**a**^ (***1***.***44***)	**5**.**43**^**b**^ (***1***.***46***)	**5**.**24**^**b**^ (***1***.***67***)

At 10 days of age, all birds were inoculated with approximately 10^5^ cfu *C*. *jejuni* KC40. A random effect was included in the statistical model at the subgroup (pen) level to account for clustering.

Most of the broilers were colonized with *C*. *jejuni* and significant differences were not observed concerning the number of colonized animals between groups. Considering *C*. *jejuni* counts in the colonized animals, mean cecal *C*. *jejuni* numbers in birds receiving hyperimmune egg yolk from bacterin-immunized layers were significantly reduced compared to birds receiving hyperimmune egg yolk from subunit vaccine-immunized layers and control broilers (resp. 4.00, 5.43 and 5.24 log_10_ cfu/g cecal content; *p*_*eq*_ = 0.015, *p* = 0.041). In birds receiving hyperimmune egg yolk from subunit vaccine-immunized layers, mean cecal *C*. *jejuni* numbers were not reduced compared to the control birds.

## Discussion

Passive immunization of broilers with hyperimmune egg yolk has previously been shown effective at reducing cecal *Campylobacter* loads when the layer hens were immunized using a whole cell lysate or its hydrophobic protein fraction^15^. In our study, a bacterin and subunit vaccine were developed for the immunization of the hens. The bacterin was composed of genetically heterogeneous *C*. *jejuni* and *C*. *coli* strains, as these two species are responsible for up to 99.6% of human campylobacteriosis cases in the EU^1^. For the subunit vaccine, proteins were selected based on the reactivity of IgY from *C*. *jejuni* KC40 immunized layer hens^15^, their association with the bacterial cell membrane^15,18–21^ and previous positive results of vaccination studies^19,22–26^. These proteins function as an ATP synthase subunit (AtpA), a chemotaxis protein associated with transmembrane receptors (CheV), an elongation factor translocated to the surface in several bacteria (EfTu), a heat shock protein shown to mediate *Salmonella* adhesion (GroEL), an amino acid transporter (LivJ) and in protein transport (Tig)^15,18–20,23–25^. In this study, the antigens proved to be highly prevalent and conserved in *C*. *jejuni*. Both vaccines could therefore be expected to offer protection against a broad range of *Campylobacter* strains *in vivo*. Immunization of hens with these vaccines resulted in a high and specific immune response, comparable to the titers obtained by Hermans *et al*.^15^. The prolonged response is an economic advantage since the hens would not need to be revaccinated during the production period.

When administered prophylactically, both treatments significantly decreased the number of *C*. *jejuni* colonized birds, particularly the bacterin-induced antibodies reduced the overall colonization rate from 78% to 15% infected chickens. The subunit vaccine treatment resulted in a reduction of the overall colonization rate to 44% infected birds. When administered therapeutically, which would be cheaper to apply in practice, the treatments were not able to significantly reduce the number of colonized birds, but the bacterin-induced antibodies were capable of significantly reducing *Campylobacter* loads in colonized animals, whereas the subunit-induced antibodies did not. These findings indicate that the bacterin-induced egg yolk antibodies yielded better results than the subunit vaccine-induced antibodies in both *in vivo* trials. Since both vaccines induced a high immune response in laying hens, the difference in protection between both vaccines cannot be attributed to insufficient antibody titers in the subunit yolks. A plausible explanation is that the bacterin contains whole cells and therefore many possible epitopes, while the subunit vaccine only contains the six selected antigens and thus a more limited number of epitopes. Bacterin-induced antibodies were previously shown to protect against *Salmonella* Enteritidis^27,28^ and *Eimeria* sp.^29,30^, but Wilkie *et al*.^31^ found no protective effect against *Clostridium perfringens*. On the contrary, earlier subunit vaccine-induced antibodies failed at protecting against *C*. *jejuni*^32^ and *Salmonella* spp.^33^.

The reduction in cecal *C*. *jejuni* titers after therapeutic administration implies that the antibodies must be active in the ceca, since the ceca were already colonized before starting the treatment. However, the site of action may not be restricted to one single gut region. Prophylactic administration possibly allows capturing the bacteria before cecal colonization, which could explain why the prophylactic model resulted in a better overall colonization reduction. Prophylactic and therapeutic passive immunization experiments with MAB carried out by Tsubokura *et al*.^14^ led to resp. a >99% and a 80–95% colonization reduction, also indicating an added value of prophylactic compared to therapeutic administration.

During colonization, *Campylobacter* can be found in the mucus layer^34^, its site of multiplication, and epithelial cells^35^, hiding from mucosal clearance^36^. Hermans *et al*.^15^ demonstrated that binding of *Campylobacter* to chicken intestinal mucus was enhanced by specific IgY. The increased bacterial uptake in the mucus layer may promote mucosal clearance, leading to the reduced colonization rates observed in our experiments.

Cross-protection for *Campylobacter* serotypes is one of the major research questions for vaccine development against *Campylobacter*, as formulated by de Zoete *et al*.^37^. The bacterin-induced antibodies strongly reacted to every bacterin and non-bacterin strain, as determined by ELISA. This indicates that passive immunization might protect against the other bacterin strains and suggests a possible cross-protection against heterologous *Campylobacter* strains, although this should be confirmed *in vivo*. Nevertheless, these antibodies seem promising at targeting a broad range of *Campylobacter* strains. In contrast, the subunit vaccine-induced antibodies reacted to the bacterin strains but only to one of the non-bacterin isolates. This can have multiple causes: (1) the genes for the subunit proteins may not be present in these strains, (2) the genes might be present but not expressed or show only a low expression rate^38^ or (3) the epitopes recognized by the antibodies might be absent or inaccessible^39^. *In vivo* protection against these strains using the subunit vaccine-derived antibodies is very unlikely, strengthening the added value of using the bacterin compared to the subunit vaccine.

In this proof of concept study, only young chickens were included in the experiments. The authors acknowledge that additional studies, including experiments in older chickens until slaughter age and field trials should be performed to support our preliminary findings.

In conclusion, two vaccines, a bacterin consisting of thirteen *C*. *jejuni* and *C*. *coli* strains and a subunit vaccine consisting of six immunodominant *Campylobacter* antigens, were developed for the immunization of laying hens. Administration of hyperimmune egg yolks induced by these vaccines to the feed of broilers, leads to a reduction of infected birds when used prophylactically and a decrease in *Campylobacter* titers when used therapeutically. Using one of both strategies, the bacterin treatment resulted in the greatest reduction. Although further research will be needed to provide a treatment protocol fully applicable in the industry, our results indicate that passive immunization of broilers with hyperimmune egg yolks of hens immunized with one of these vaccines, especially the bacterin, offers possibilities to control *Campylobacter* colonization in poultry.

## Methods

### Experimental animals

Commercial Lohmann Brown-Classic laying hens, LSL-Classic laying hens and Ross 308 broiler chickens of both sexes were purchased at a local hatchery (layers at De Biest, Kruishoutem, Belgium and broilers at Vervaeke-Belavi, Tielt, Belgium). The animals were provided with a commercial feed and water *ad libitum*. Husbandry, experimental procedures, euthanasia methods and bio-safety precautions were approved by the Ethical Committee (EC) of the Faculty of Veterinary Medicine, Ghent University, Ghent, Belgium (EC number: 2016/28) and in accordance with the relevant guidelines and regulations. Birds were proved to be free of *Campylobacter* by examination of mixed fecal samples using standard methods as described by Hermans *et al*.^40^.

### Bacterial strains and culture conditions

The *Campylobacter* strains used in this study are listed in Table 5. For all experimental infections in the *in vivo* trials, *C*. *jejuni* reference strain KC40 from poultry origin was used, which colonizes chickens to a high level^36^. For bacterin composition, *Campylobacter* strains were kindly provided by Dr. Nadine Botteldoorn (Sciensano, Brussels, Belgium), except for the *C*. *jejuni* KC40 reference strain which was previously isolated at the Flanders Research Institute for agriculture, fisheries and food (ILVO, Melle, Belgium). The strains are from chicken origin and were selected based on their genetic heterogeneity based on multilocus sequence typing (MLST), prevalence ratio in broilers^16^ and relationship with human campylobacteriosis cases^41^. The remaining *Campylobacter* strains, used for ELISA crossreaction studies, are from chicken origin and were selected based on their genetic heterogeneity and distinction from the bacterin strains using MLST^42^.

**Table 5:** *C*. *jejuni* and *C*. *coli* strains from chicken origin used in this study.

*Campylobacter* species	Strain	CC	ST	Origin
*C*. *jejuni*	KC40^b^	677	794	Broiler dunghill
	10kf-1.16^b^	283	267	Carcass
	7P6.12^b^	464	464	Feathers
	10C-6.1^b^	574	305	Ceca
	10kf-4.12^b^	443	51	Carcass
	10VTDD-8^b^	UA	905	Unknown
	T124^b^	658	1044	Ceca
	T84^b^	354	1073	Ceca
	T70^b^	21	50	Carcass
	3291^b^	45	45	Carcass
	5970^b^	UA	5970	Carcass
	5CT13	48	429	Ceca
	3CT13	52	600	Ceca
	1CT117	257	5742	Ceca
	1CT51	353	462	Ceca
*C*. *coli*	2711^b^	828	854	Carcass
	3250^b^	UA	5163	Carcass

CC: Clonal complex; ST: Sequence type; ^b^strains incorporated in the bacterin; UA: Unassigned.

Bacteria were routinely cultured in Nutrient Broth No. 2 (NB2, CM0067; Oxoid Ltd., Basingstoke, Hampshire, UK) supplemented with Modified Preston *Campylobacter*-selective supplement (SR0204E; Oxoid) and *Campylobacter*-specific growth supplement (SR0232E; Oxoid), at 42 °C for 17 h under microaerobic conditions (5% O_2_, 5% CO_2_, 5% H_2_, 85% N_2_). *C*. *jejuni* and *C*. *coli* bacteria were enumerated by plating tenfold dilutions in Hank’s Balanced Salt Solution (HBSS; GIBCO-BRL, Invitrogen, Carlsbad, CA) on modified charcoal cefoperazone deoxycholate agar (mCCDA, CM0739; Oxoid) supplemented with CCDA selective supplement (SR0155E; Oxoid) and *Campylobacter*-specific growth supplement (SR0232E; Oxoid), followed by microaerobic incubation at 42 °C for 22 h.

### Prevalence and conservation level of immunodominant *Campylobacter* antigens

Based on the results of Hermans *et al*.^15^, six immunodominant antigens with high reactivity to IgY from eggs of chickens immunized against *C*. *jejuni* were selected: AtpA, Ef-Tu, GroEL, Tig, CheV and LivJ. These proteins are known or suggested to be expressed on the bacterial cell surface (EfTu, GroEL) or known to be associated with the cell membrane (AtpA, CheV, LivJ, Tig)^15,18–21^. Previously, positive results were obtained when vaccinating with these proteins^19,22–26^.

The prevalence and the conservation level of the genes coding for these immunodominant proteins were determined in the *Campylobacter* strains selected for constructing the bacterin using PCR. Because of the genetic heterogeneity, separate primers were developed for *C*. *jejuni* and *C*. *coli* strains (http://www.ncbi.nlm.nih.gov/gene/) (Table S1, Supplementary Materials). *Campylobacter* strains were plated on Columbia Sheep Blood agar (CSB, Oxoid) and incubated overnight at 37 °C under microaerobic conditions (5% O_2_, 5% CO_2_, 5% H_2_, 85% N_2_). For DNA extraction, colonies were incubated with 20 µL lysis buffer (1/40 10% SDS, 1/20 1N NaOH in AquaDest) until the formation of slime was visible, and afterwards incubated at 95 °C for 10 min. After cooling to condense the water vapor and short centrifugation, 80 µl high performance liquid chromatography (HPLC, Merck, VWR, Amsterdam, Netherlands) grade water was added. The lysate was centrifuged at 13000 rpm for 5 min and the supernatant was stored at −20 °C. The amplification of DNA was performed in a Mastercycler (Eppendorf AG, Hamburg, Germany) in a volume of 25 µL with 1X mastermix [dNTP’s, MgCl and NA polymerase of Bioline (Luckenwalde, Germany)] and 0.5 µM of each primer. *C*. *jejuni* strain KC40 was used as a positive control and blanc HPLC water was added to the mix as a negative control. The PCR program was set at 4 min at 95 °C, 35 cycles (1 min at 94 °C, 1 min at 57 °C, 1 min 30 s at 72 °C) and a final elongation step of 15 min at 72 °C. The PCR reaction products were analyzed with gel electrophoresis. Sequencing analysis was performed to determine the degree of conservation of the prevalent encoding proteins. For genes consisting of more than 1000 base pairs, multiple primer pairs were developed (Table S1). The DNA amplification and gel electrophoresis were performed as described above. After checking the purity of the bands, sequencing analysis was performed by Eurofins Genomics (Ebersberg, Germany). Data were analyzed using Nucleotide BLAST (https://blast.ncbi.nlm.nih.gov/Blast) for comparison of the nucleotide sequences, ExPASy Bioinformatics Resource Portal (http://web.expasy.org/translate/) to translate the nucleotide sequences into protein sequences and Protein BLAST (https://blast.ncbi.nlm.nih.gov/Blast) for comparison of the protein sequences.

### Preparation of recombinant *C*. *jejuni* antigens

For recombinant production of the immunodominant antigens, derived from the *C*. *jejuni* reference strain KC40, the *E*. *coli* Expression System using Gateway® Technology (Invitrogen) was used. Signal peptides in the coding regions, which were screened by using the SignalP 4.1 server (http://www.cbs.dtu.dk/services/SignalP/), were removed. The coding regions were then amplified by PCR, using Pwo polymerase with proofreading activity (Roche Applied Science, Mannheim, Germany) according to the manufacturer’s instructions and with the primers given in Table S1. The resulting PCR products were cloned into the pENTR™/TEV/D-TOPO® vector (AtpA, EF-Tu and GroEL) or the pENTR™/SD/D-TOPO® vector (Tig, CheV and LivJ) using the Topo TA cloning kit (Invitrogen) according to the manufacturer’s instructions. Next, the genes were transferred into the pDEST™17 destination vector and the resulting expression clones were transformed into BL21-AI One Shot® chemocompetent *E*. *coli* cells (Invitrogen).

A fresh transformed *E*. *coli* culture was grown in 100 mL Luria Broth medium (LB, Oxoid) supplemented with 50 µL/mL carbenicillin at 37 °C with shaking until an OD_600_ of 0.6–1.0 was reached. The culture was inoculated in 6 × 200 mL fresh LB medium supplemented with 50 µL/mL carbenicillin at an OD_600_ of 0.05–0.1 and grown at the same circumstances until an OD_600_ of 0.4 was obtained. Next, 0.2% L-arabinose was added to induce expression of the recombinant antigens. After 6 h of incubation, the cultures were centrifuged (30 min, 4500 rpm) and the pellets were resuspended in binding buffer (40 mM imidazole, 10 mL binding buffer per 1 g pellet). Next, 100 µL lysozyme (20 µg/ml), 200 µl DNase (Sigma Aldrich, Steinheim, Germany), 50 µl 200 × MgCl_2_ and 100 µl protease inhibitor (Sigma) were added and the mixture was shaken (30 min). After sonication (7x, 15 sec, maximal amplitude), the lysate was centrifuged (30 min, 4500 rpm). The supernatant was purified on Ni-sepharose columns (His GraviTrap; GE Healthcare Bio-science AB, Uppsala, Sweden) according to the manufacturer’s instructions. Bound proteins were eluted with 3 mL elution buffer (20 mM sodium phosphate, 500 mM NaCl, 500 mM imidazole, pH 7.4) and collected in 17 mL HBSS. The eluate was concentrated to a final volume of 1.5 mL using ultrafiltration (VIVASPIN 20, 5000 MCWO; Sartorius Stedem Biotech, Goettingen, Germany) and analyzed by sodium dodecyl sulfate polyacrylamide gel electrophoresis (SDS-PAGE), followed by Brilliant Blue G-Colloidal (Sigma) coloring and Western blotting.

For the Western blot, separated proteins were electrotransferred from SDS-PAGE gels onto nitrocellulose membranes (Bio-Rad, Nazareth, Belgium) as described previously^43^. Membranes were blocked in 5% skimmed milk in phosphate buffer saline (PBS) (blocking buffer), incubated overnight with mouse monoclonal antibody to hexahistidine tag (1/3000 in blocking buffer, Icosagen Cell Factory, Tartu, Estonia) at room temperature (RT), rinsed in PBS with 0.3% Tween-20 (wash buffer) and incubated for 1 h at RT with rabbit anti-mouse IgG (whole molecule)–peroxidase antibody (1/30 000 in blocking buffer, Sigma-Aldrich). After a wash step in wash buffer, 10 × CN/DAB Concentrate in Stable Peroxide Substrate Buffer (Thermo Scientific) was added for immunodetection of proteins. Protein patterns were scanned using the GS-800 Calibrated Densitometer (Bio-Rad). The protein concentrations were determined using the *RC DC* Protein Assay (Bio-Rad) and the purified proteins were stored at −80 °C until further use.

### Bacterin and subunit vaccine preparation

The bacterin was composed as follows: 13 *Campylobacter* strains (Table 5) were grown separately in NB2 until 9 log_10_ colony forming units (cfu)/mL and killed by overnight incubation with 5 mL 36% formaldehyde/L (Sigma-Aldrich) at 37 °C. After centrifugation (30 min at 5000 rpm at 20 °C), the pellets were resuspended in 5 mL 36% formaldehyde/L PBS and incubated overnight at 37 °C. After plating on CSB agar and overnight incubation at 37 °C to check that all the cells were killed, the suspensions were stored at 4 °C. A mix of the 13 *Campylobacter* suspensions was made, so that each bacterin dose consisted of 8.1 log_10_ cfu inactivated *Campylobacter* (i.e. 7 log_10_ cfu/*Campylobacter* strain).

For the subunit vaccine, 75 µg protein (i.e. 12.5 µg of each recombinant antigen) was supplemented with HBSS until a volume of 125 µL/vaccine dose. For sham immunization, 125 µL HBSS was used (negative control).

Each immunization dose consisted of 250 µL of a 1:1 mixture of the inoculum with Freund’s Complete Adjuvant (FCA, Sigma-Aldrich) for the first immunization and Freund’s Incomplete Adjuvant (FIA, Sigma-Aldrich) for the boosters.

### Immunization of layers

Thirty *Campylobacter*-free commercial Lohmann Brown-Classic (LBC) and thirty Lohmann LSL-Classic (LLC) layer hens were assigned to the following immunization groups at the age of 20 weeks: bacterin (n = 20 LLC hens), subunit (n = 20 LBC hens) and control (n = 10 LLC hens; n = 10 LBC hens). Chickens were immunized by intramuscular injection in the pectoral muscle with the vaccines composed as described above. Three booster immunizations were given in a two-weekly time interval. Starting from one week after the last immunization, eggs were collected and stored at 4 °C.

### Determination of egg yolk IgY titers

*Campylobacter*-specific IgY titers in egg yolks were determined as previously described by Hermans *et al*.^15^ with minor changes to the protocol. Egg yolks were diluted 1/5 (vol/vol) in HBSS, mixed thoroughly and incubated overnight at 4 °C. The supernatant, containing the water-soluble fraction of the egg yolk, was collected for IgY quantification using enzyme-linked immunosorbent assay (ELISA). To determine egg yolk IgY titers against the complete bacterin and the complete subunit vaccine, 96 well flat bottom plates (Nunc MaxiSorp, Nalge Nunc Int., Rochester, NY, USA) were coated (24 h, 4 °C) with 10^6^ cfu bacterin or 3 µg of a mixture of subunit antigens diluted in 50 µL coating buffer (2.16 g Na_2_CO_3_.10H_2_O, 1.935 g NaHCO_3_ in 500 mL H_2_O). To determine egg yolk IgY titers against each recombinant antigen, separately, plates were coated with 3 µg of AtpA, CheV, EfTu, GroEL, LivJ or Tig diluted in 50 µL coating buffer. To determine egg yolk IgY titers against the different *Campylobacter* strains, plates were coated with 10^6^ cfu/strain, diluted in 50 µL coating buffer. After washing (3x HBSS, 1x washing buffer: 0,1% Tween-20 in PBS), the wells were blocked (1 h, room temperature) with 100 µl blocking buffer [1% bovine albumin serum (BSA) in washing buffer]. Next, 100 µL of a 1/2 dilution series of the supernatant of the mixed egg yolks was incubated during 60 min at room temperature. Plates were washed as described above and incubated with 100 µL 1/10,000 horseradish peroxidase (HRP)-labelled anti-chicken IgY (Sigma Aldrich) in washing buffer during 90 min at room temperature. After washing as described above, the plates were incubated with 50 µl 3,3′,5,5′-tetramethyl benzidine (TMB) substrate (Sigma Aldrich) for 10 min at room temperature in the dark. Next, 50 µL 0.5 M H_2_SO_4_ was added to each well and the absorbance at 450 nm (OD_450_) was measured using an automated spectrophotometer (Pharmacia LKB Ultrospec III, Gemini BV, Apeldoorn, Nederland). The IgY titers from yolks of immunized hens were reported as the highest dilution where the OD_450_ was greater than the OD_450_+ three standard deviations of wells containing yolk originating from sham vaccinated birds^15^.

### Prophylactic efficacy of in-feed supplementation of bacterin and subunit vaccine derived hyperimmune egg yolk on transmission of and cecal colonization with *C*. *jejuni* in broilers

In trial 1, 81 day-of-hatch *Campylobacter* free broilers were raised in three randomly assigned treatment groups (n = 27/group) and housed in separate isolation units. From the day of hatch until the end of the experiment, the chicks were provided with feed containing 5% (wt/wt) egg yolk (mixed manually through the feed) from hens immunized with the bacterin (group 1), subunit vaccine (group 2) or sham-immunized with HBSS (group 3). Equal amounts of feed and drinking water were provided for each group during treatment and care was taken that all animals had unlimited access to the feed and water. At 10 days of age, the chicks of each group were randomly assigned to three subgroups (n = 9/subgroup) and housed in separate isolation units. At 11 days of age, three seeder chicks of each subgroup were randomly selected and orally inoculated with approximately 1 × 10^5^ cfu of *C*. *jejuni* strain KC40. The birds that were not inoculated are referred to as contact animals or sentinels. Using this model, the *Campylobacter* infection will spread from the seeders to the other animals of the same group reproducing the natural way of infection in the stable and prevention of infection and transmission can be investigated^15^. At day 16, all animals were euthanized by injection of an overdose (100 mg/kg) sodium pentobarbital (Kela, Hoogstraten, Belgium) in the wing vein and the cecal content was collected for *C*. *jejuni* enumeration (as described below).

### Therapeutic efficacy of in-feed supplementation of bacterin and subunit vaccine derived hyperimmune egg yolk on cecal *C*. *jejuni* colonization in broilers

In trial 2 a therapeutic model was used to test the effect of treatments in birds already colonized with *Campylobacter*. For this, 81 day-of-hatch *Campylobacter* free broilers were raised in three randomly assigned groups (n = 27/group) and housed in separate isolation units. At 9 days of age, the chicks of each group were randomly assigned to three subgroups (n = 9/subgroup) and housed in separate isolation units. At 10 days of age, all chicks were orally inoculated with approximately 1 × 10^5^ cfu of *C*. *jejuni* strain KC40, similar to the inoculation during the first trial. From day 19 to 21, the chicks were provided with feed containing 5% (wt/wt) egg yolk (mixed manually through the feed) from hens immunized with the bacterin (subgroups 1, 2, 3), subunit vaccine (subgroups 4, 5, 6) or sham-immunized with HBSS (subgroups 7, 8, 9). Since the therapeutic effect on colonized broilers was to be investigated, all birds were inoculated and sufficient time was given between inoculation and the beginning of the treatment to obtain high *Campylobacter* titers in the gut, comparable to the field situation. At day 22, all animals were euthanized (as described above) and the cecal content was collected for *C*. *jejuni* enumeration (as described below).

### Cecal Campylobacter jejuni enumeration

Cecal contents were weighed and diluted 1:9 (wt/vol) in NB2 with supplements. A 10-fold dilution series was made in HBSS and 100 µl of each dilution was spread on mCCDA plates. Colonies were counted after 24 h and 48 h incubation at 42 °C under microaerobic conditions. The diluted samples in NB2 were incubated overnight at 42 °C under microaerobic conditions for enrichment. Samples were plated on mCCDA and further incubated. After 24 h and 48 h, the plates were examined for the presence or absence of *C*. *jejuni*. Samples negative after titration and enrichment were considered to be free of *Campylobacter* (<10^2^ cfu/g cecal content, limit of detection). Samples negative after titration but positive after enrichment were considered to contain 10^2^ cfu/g cecal content.

### Statistical analysis

Data of the *in vivo* trials were analyzed using R 3.3.1. Before statistical analysis, *C*. *jejuni* counts were transformed to log_10_ counts. The colonization data were analyzed using a hurdle model^44,45^, a class of model that assumes that the data are generated by two processes. First, the event that an individual is colonized (i.e. returning a non-zero count) follows a Bernoulli distribution. Given colonization, its intensity or load is a random variable following a discrete or continuous distribution; in this case, a gamma distribution was assumed.

The influence of treatment was assessed by specifying predictors for the Bernoulli probability of occurrence (i.e. probability of colonization, modelled as a logistic function of covariates) and the rates of the gamma distribution (average *C*. *jejuni* counts given colonization, modelled as a log-linear function of covariates). In both functions treatment was included as a categorical covariate (bacterin/subunit/control). The sample size prevented the inclusion of an additional covariate for individual type (seeder/sentinel) and the associated interaction term for the first *in vivo* trial. Instead, the analysis was repeated for all birds and for seeders and sentinels separately. A random effect was included at the subgroup (pen) level to account for clustering.

The model was implemented in a Bayesian framework using JAGS^46^. Uninformative, flat priors were used for all parameters. Over three Markov chains, 100.000 iterations were run, discarding the first 50.000 as a burn-in. Convergence was assessed by visual inspection of the chain histories and using the Gelman-Brooks-Rubin statistic^47^. The model was used to estimate the probability of *C*. *jejuni* colonization and the mean *C*. *jejuni* numbers in the cecal content of colonized birds for each treatment level. Next, the pairwise differences between those, and the proportion of the respective posterior distributions that had the same sign as the mean were calculated. If working in a null-hypothesis significance testing framework, this can be interpreted as a one-sided test (broilers treated with bacterin-induced antibodies versus control birds, broilers treated with subunit vaccine-induced antibodies versus control birds), estimating the probability that the true difference between treatments is zero or greater (if negative) or smaller (if positive), and thus the level of confidence that the null hypothesis can be rejected. The broilers treated with bacterin- and subunit vaccine-induced antibodies were compared with the equivalent of a two-sided test; the null hypothesis was retained when the posterior distribution of the difference did not encompass zero between the 2.5% and 97.5% quantiles.

## Supplementary information

Supplementary info

## Data Availability

The datasets generated during the current study are available from the corresponding author on reasonable request.
