# Supplementary material for: Reducing Campylobacter jejuni colonization in broiler chickens by in-feed supplementation with hyperimmune egg yolk antibodies
Source: Sci Rep. 2019 Jun 20;9:8931. doi: 10.1038/s41598-019-45380-z (PMC6586802; doi:10.1038/s41598-019-45380-z)
Supplement: Supplementary file 1 — Supplementary info [file 41598_2019_45380_MOESM1_ESM.pdf]

Reducing *Campylobacter jejuni* colonization in broiler chickens by in-feed supplementation with hyperimmune egg yolk antibodies

Jasmien Vandeputte[a], An Martel[a], Stefano Canessa[a], Nathalie Van Rysselberghe[a], Lieven De Zutter[b], Marc Heyndrickx[a,c], Freddy Haesebrouck[a], Frank Pasmans[a] and An Garmyn[a]

[a]Department of Pathology, Bacteriology and Avian Diseases, Faculty of Veterinary Medicine, Ghent University, Salisburylaan 133, B9820 Merelbeke, Belgium

[b]Department of Veterinary Public Health and Food Safety, Faculty of Veterinary Medicine, Ghent University, Salisburylaan 133, B9820 Merelbeke, Belgium

[c]Flanders Research Institute for Agriculture, Fisheries and Food (ILVO), Technology & Food Sciences Unit, Brusselsesteenweg 370, B9090 Melle, Belgium

SUPPLEMENTARY NOTES

We have developed two novel vaccines, a bacterin and subunit vaccine, and used the vaccines to protect broiler chickens against *Campylobacter* colonization by passive immunization. Posterior distributions from the statistical analysis of the *in vivo* trials' results and primers used for vaccine development can be found in the supplementary materials.

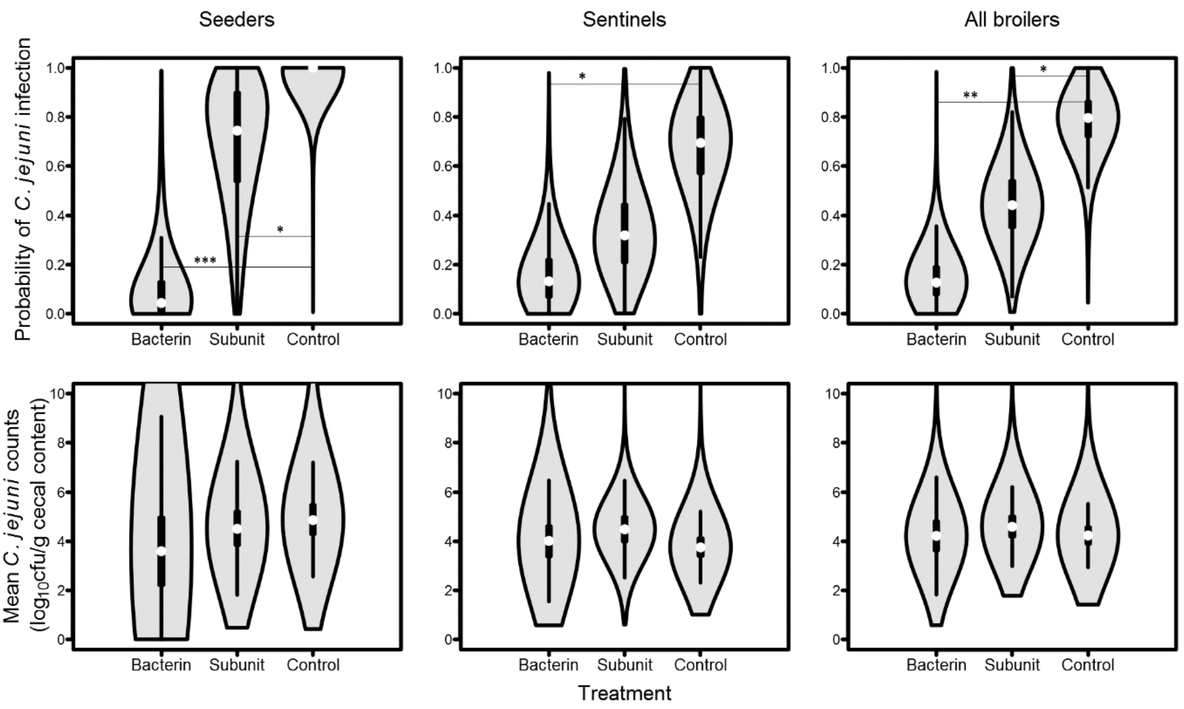

Figure S1. Posterior distributions of the estimated probabilities of *C. jejuni* colonization (top) and mean *C. jejuni* numbers in the cecal content of colonized birds (bottom) for seeders, sentinels or all broilers after prophylactic treatment. The distributions are visualized by a boxplot and Gauss curve. The birds received standard feed supplemented with 5% (wt/wt) egg yolk from either bacterin-immunized (Bacterin), subunit vaccine-immunized (Subunit) or sham-immunized (Control) layers, from day 1 until day 16 (the day of euthanasia). At 11 days of age, seeder birds were inoculated with approximately 10^5 cfu *C. jejuni* KC40.

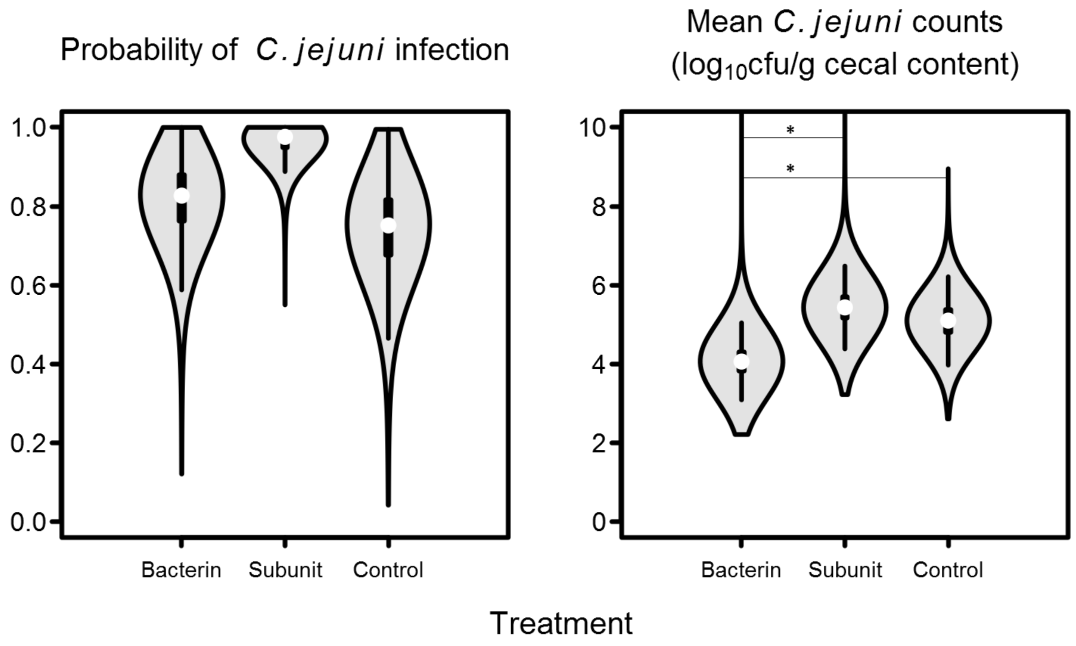

Figure S2. Posterior distributions of the estimated probabilities of *C. jejuni* colonization (left) and mean *C. jejuni* numbers in the cecal content of colonized birds (right) after therapeutic treatment. The distributions are visualized by a boxplot and Gauss curve. The birds received standard feed supplemented with 5% (wt/wt) egg yolk from either bacterin-immunized (Bacterin), subunit vaccine-immunized (Subunit) or sham-immunized (Control) layers, from day 19 until day 21 (the day of euthanasia). At 10 days of age, all birds were inoculated with approximately 10^5 cfu *C. jejuni* KC40.

Supplementary table S1. Primers used for screening of the *Campylobacter* bacterin strains for the presence of immunodominant antigens, sequencing of the prevalent immunodominant antigens and production of blunt end PCR products of the immunodominant antigens

Gene fragment	Forward primer	Reverse primer	Fragment length (bp)
AtpA			
Presence in *C. jejuni*	5'-AGCTGATGAGATCAGTTC-3'	5'-AGCGGAGAATAAGGTGGTTG-3'	1271
Presence in *C. coli*	5'-AGCTGATGAAATCAGTTC-3'	5'-AGTGGAGAATAAGGAGGTTG-3'	1271
Sequencing fragment 1	5'-GAAAGCTCTTCAAAATAAGC-3'	5'-AGACATTTCGCGATAAGC-3'	884
Sequencing fragment 2	5'-GCTCCATATACTGGTGTAACC-3'	5'-CTTTTAGCTGC TTCTTCTGC-3'	925
Production of blunt end PCR products	5'-CACCATGAAATTTAAAGC-3'	5'-TTATAAATGATTTGCTTTAAACTC-3'	1505
CheV			
Presence in *C. jejuni*	5'-ATGGAGCTTGTCGATTTCCG-3'	5'-TTACCCCTGTTCTTGAGATTG-3'	915
Presence in *C. coli*	5'-ATGGAGCTTGTCGATTTCCG-3'	5'-TTACCCCTGTTCTTGAGATTG-3'	915
Sequencing fragment	5'-TGGGTATGTGAAGGACAC-3'	5'-GTCGATTTGCTCAACAAGC-3'	1183
Production of blunt end PCR products	5'-CACCATGTTTGATGAAAATATCG-3'	5'-TTACCCCTGTTCTTGAGATTG-3'	957
Ef-Tu			
Presence in *C. jejuni*	5'-CACGTAATAAGCCACACG-3'	5'-ACCACCTTCACGAATAGC-3'	1138
Presence in *C. coli*	5'-CACGTAATAAGCCACATG-3'	5'-ACCACCTTCACGGATAGC-3'	1138

Sequencing fragment 1	5'-TTTCTGAGCGCTCGTATGGC-3'	5'-CCTGTAACAACAGTACCACG-3'	855
Sequencing fragment 2	5'-CTCTTATGATTTCCCAGGCG-3'	5'-TATTGCCCTAAGGGCGCAAGC-3'	955
Production of blunt end PCR products	5'-CACCATGGCTAAAGAAAA-3'	5'-TTATTTAATAATTTTAGAAAC-3'	1199
GroEL			
Presence in *C. jejuni*	5'-CAGGCGATGGAACAACTACTGC-3'	5'-CCATACCGCTCATATCTGGC-3'	1356
Presence in *C. coli*	5'-CAGGCGATGGAACAACTACTGC-3'	5'-CCATACCGCTCATATCTGGC-3'	1356
Sequencing fragment 1	5'-GCTAAATACGGTGGAACAG-3'	5'-CGCTATATCTTCAAGCATAGC-3'	983
Sequencing fragment 2	5'-CGCTGAAGATATTGAAGG-3'	5'-GAGGATTTGGTATAGGGC-3'	1047
Production of blunt end PCR products	5'-CACCATGGCAAAAGAAAT-3'	5'-TTACATCATTCCGCCCATGC-3'	1637
LivJ			
Presence in *C. jejuni*	5'-CTTTAACTGGAACTGTGGCAGC-3'	5'-GCATTACCGCTTTCATCTATGC-3'	961
Presence in *C. coli*	5'-CATTAACAGGACCAGTGGCTGC-3'	5'-GCATTACCGCTTGCATCAATGC-3'	961
Sequencing fragment 1	5'-GCAGTTCTACAACAGCTTCT-3'	5'-TTGTTAACACCATCTCCTGC-3'	949
Sequencing fragment 2	5'-GCTCCAGTGGCATCTGGAGATA-3'	5'-TGTCCATATGCTGCAGTAGC-3'	902
Production of blunt end PCR products	5'-CACCATGAAGGATATTAATATAGG-3'	5'-TTATGGATTTATAATTGTTTTATA-3'	1115
Tig			
Presence in *C. jejuni*	5'-GGCAAAGCAACTAGATTCTG-3'	5'-TCACAGCAGGCAAAGCTCCTTG-3'	1226

Presence in *C. coli*	5'-AGCAAAGCAATTAGACTCTG-3'	5'-TCACAGCAGGCAAAGCTCCTTG-3'	1226
Sequencing fragment 1	5'-AAGCCCTTCACGATTTGG-3'	5'-TACTGCATCTTTACCCGC-3'	925
Sequencing fragment 2	5'-CGTTTTGCTACTCCTGAAGC-3'	5'-GTCATAACTTCTTTCACCACG-3'	994
Production of blunt end PCR products	5'-CACCATGGAAGTAAAGGC-3'	5'-TTATTTATCTTCTTTCTC-3'	1330
